# Low diagnostic performance of thick blood smears of 50 µl in comparison with direct examination of 10 µl blood and the leukoconcentration technique of 5ml blood among loiasis-suspected patients with low microfilaremia in Gabon, Central Africa, using the STARD-BLCM guidelines

**DOI:** 10.1186/s13071-023-06089-1

**Published:** 2024-03-15

**Authors:** Noé Patrick M’Bondoukwé, Matthieu Owono-Medang, Marie Noëlle Moussavou-Boussougou, Yvan Akoue, Valentin Migueba, Dmitry Bulaev, Anouk Neven, Luice Aurtin Joel James, Sylvie Alberte Ntsame Ella, Denise Patricia Mawili-Mboumba, Julienne Atsame, Michel Vaillant, Marielle Karine Bouyou Akotet

**Affiliations:** 1 Department of Parasitology-Mycology-Tropical Medicine, Faculty of Medicine, Université des Sciences de la Santé, 4009 Libreville, Gabon; 2 Programme de Lutte Contre les Maladies Parasitaires, Ministère de la Santé du Gabon, Libreville, Gabon; 3 Competence Centre for Methodology and Statistics, Luxembourg Institute of Health, 1A-B Rue Thomas Edison, 1445 Strassen, Luxembourg

**Keywords:** Loiasis, Diagnostic accuracy, Direct blood examination of 10 µl, Thick blood smear of 50 µl, Leukoconcentration technique of 5 ml, STARD-BLCM

## Abstract

**Background:**

The aim of this study was to determine performance indicators of thick blood smears of 50 µl (TBS-50), following the Standards for the Reporting of Diagnostic Accuracy Studies–Bayesian Latent Class Model (STARD-BLCM) guidelines. TBS-50 was compared with two common parasitological techniques—direct examination of 10 µl blood and a leukoconcentration of 5 ml—for the diagnosis of microfilaremic loiasis.

**Methods:**

The study population was recruited among patients of the Department of Parasitology-Mycology-Tropical Medicine over a period of 1 year. Age, sex, symptoms, and eosinophilia variables were recorded from laboratory registers and medical files. Direct examination of 10 µl of blood, TBS-50, and the leukoconcentration technique with 5 ml of blood were performed for each patient. The classical formula and BLCM were used to determine the diagnostic accuracy of the three techniques as well as the prevalence of microfilaremic loiasis. Three models were built within the framework of BLCM—the BLCM model I and alternative models II and III—for sensitivity analysis.

**Results:**

In total, 191 patients consented to be included. The direct blood examination and TBS-50 yielded comparable qualitative and quantitative results. Hence, they are reported together. The prevalence of *Loa loa* microfilaremia was 9.4% (95% CI 5.7–14.5; *n* = 18/191) with direct blood examination/TBS-50 and 12.6% [8.2–18.1] (*n* = 24/191) for leukoconcentration. Comparing TBS-50 with the leukoconcentration method using the classical formula, the sensitivity was 75.0% [53.3–90.2], specificity was 100.0% [97.8–100.0], the positive predictive value was 100.0% [81.5–100.0], and the negative predictive value was 96.5% [92.6–98.7]. The prevalence of microfilaremic loiasis was estimated at 9.7% [6.2–13.7] using BLCM model I. The outputs of BLCM model I showed sensitivity of 78.9% [65.3–90.3], specificity of 100.0% [99.3–100.0], a positive predictive value of 99.1% [87.2–100.0], and a negative predictive value of 93.0% [87.3–97.7] for direct blood examination/TBS-50.

**Conclusions:**

TBS-50 demonstrates low sensitivity relative to two other techniques. In one in five cases, the result will be falsely declared negative using these methods. However, this method can be deployed with limited funds.

**Graphical Abstract:**

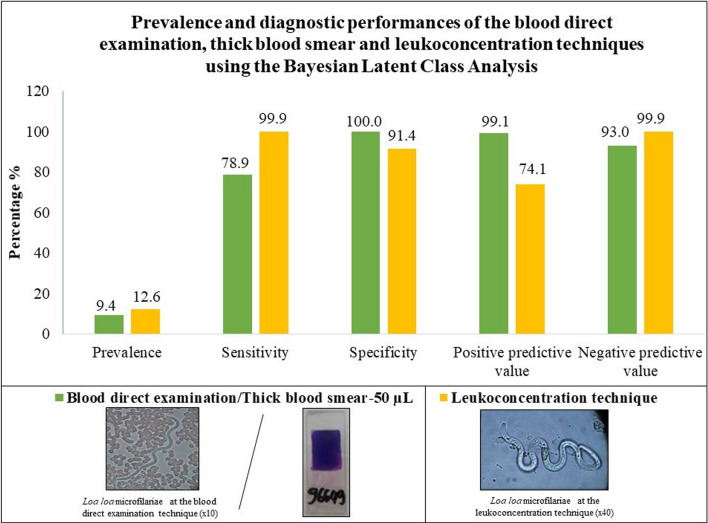

**Supplementary Information:**

The online version contains supplementary material available at 10.1186/s13071-023-06089-1.

## Background

Loiasis, a vector-borne disease caused by the parasitic worm *Loa loa*, is a common reason for medical consultation in Gabon, with prevalence varying between 6.0 and 40.0%, depending on the area [1–3]. This filarial parasitosis is found in forest areas where the *Chrysops* vector lives, particularly in rural areas. In urban areas it is possible to see some patients who have been infected, but as a result of previous exposure in rural areas [4]. Clinical signs may include symptoms such as Calabar swelling, eye worms, and the sensation of crawling under the skin, as well as pruritus, itching, and urticaria. A recent study has shown that infected individuals may also suffer from atypical manifestations such as cardiac, respiratory, gastrointestinal, renal, neurological, ophthalmological, and dermatological disorders [5]. From an immunological point of view, an increased level of polynuclear eosinophils (hypereosinophilia) may be observed with values greater than or equal to 7.0% of total white blood cells [6].

In many regions in Gabon, loiasis is co-endemic with onchocerciasis, one of the neglected tropical diseases (NTDs) targeted to be eliminated by 2030. Generally, co-endemicity with loiasis prevents elimination of onchocerciasis but also of non-endemic lymphatic filariasis. Indeed, following diethylcarbamazine (DEC) and ivermectin (IVM) mass drug administration for the treatment of onchocerciasis, severe adverse events (SAEs) were reported, leading to functional impairment that may be reversible, as well as encephalopathy-caused death among patients with high *L. loa* microfilaremia (> 8000 microfilariae [mf]/ml) [7, 8]. The unselective use of these drugs on a large scale may cause lysis of the *L. loa* microfilariae, inducing SAEs due to the strong inflammatory response. Integrated cartography identifying co-endemic areas is therefore important because it enables the deployment of appropriate treatment strategies [9]. Moreover, in a recent study, Chesnais et al. found that the mortality risk increases in populations with levels of microfilariae exceeding 30,000 mf/ml [10]. Hypermicrofilaremia is often detected in rural areas [11, 12]. Therefore, the availability of a powerful diagnostic tool that would be easy to use in landlocked areas is of major interest in loiasis management. Indeed, as the road network in Gabon is degraded, populations far from towns are isolated from health facilities. Thus, the use of an alternative diagnostic test able to determine parasite density as precisely as possible is critical in order to classify patients at risk of SAEs.

Conventional loiasis laboratory diagnostics are performed using a light microscope with direct examination of 10 μl blood samples coupled with leukoconcentration analysis of 4–5 ml of blood [13]. There are other diagnostic tools, such as LoaScope, with 100.0% sensitivity and 94.0% specificity when compared with a TBS [14]. This test was developed to quickly diagnose (~2 min) hypermicrofilaremic loiasis in rural areas during mass drug administration with IVM, where no technical platform is available for the realization and reading of conventional techniques. Patients with low microfilaremia or without microfilaremia can be safely administered IVM/DEC without the occurrence of SAEs. However, this technology has limited availability [15]. The loop-mediated isothermal amplification (LAMP) technique is increasingly being developed and evaluated for the rapid molecular diagnosis of loiasis. This technique has shown sensitivity of approximately 90.0% when compared with microscopy and polymerase chain reaction (PCR)-based assays [16]. It can provide information both on the presence and absence of microfilariae and on the microfilaremia without the need to use microscopy, with colored reaction [17, 18].

In Gabon, conventional techniques are performed in some specialized medical institutions [1–3]. The limit of detection is generally set to 100 mf/ml for direct examination and to 1 mf/ml for the leukoconcentration technique to detect low-density carriers. To the best of our knowledge, no published data are available on the diagnostic performance of these tests. In remote areas of low- and middle-income countries, their implementation is challenging in screening campaigns due to the lack of electrical infrastructure. To overcome this challenge, a thick blood smear (TBS) was proposed as a large campaign survey method by Sasa [19]. In Gabon, national cartography was performed in 2014–2015 for loiasis, onchocerciasis, soil-transmitted helminthiasis, schistosomiasis, and human trypanosomiasis. The technique used for loiasis identification was a calibrated TBS of 50 μl (TBS-50) from blood capillary collection. It offers the advantage of collecting blood samples in the field and then analyzing them later in a clinical laboratory. This same technique was used in Cameroon and the Democratic Republic of the Congo, two loiasis-endemic countries of Central Africa located to the north and west of Gabon, respectively [11, 20–25].

According to recent epidemiological data, nearly 30.0% of the infected population present microfilaremic loiasis, and almost 5.0% have hypermicrofilaremia (> 8000 mf/ml) [23, 26, 27]. It has been shown that patients with symptoms such as eye worm or Calabar swelling generally have low parasite density, undetectable with the direct examination of 10 μl of blood [1]. The TBS-50 technique is assumed to be more sensitive, with five times the volume of blood in the direct examination and with a much lower limit of detection of 20 mf/ml, which would enable more efficient management of patients with symptoms but in whom the direct examination failed to identify microfilariae carriers. However, no data in the literature are equally available on the diagnostic accuracy of this technique. Sensitivity of only 67.0% and 62.0% and negative predictive value (NPV) of 93.0% and 74.0% were reported for *L. loa* and *Mansonella perstans*, respectively, for a TBS of 20 μl × 2, in comparison with saponin hemolysis of 5 ml of blood [28].

The aim of this study was to estimate the performance indicators of direct examination of 10 µl of blood, TBS-50, and the leukoconcentration technique using classical formulas and a Bayesian latent class model (BLCM). Classical formulas assume perfect gold standards. Bayesian latent class analysis (BLCA) is commonly used when there is no “perfect” test for disease diagnosis available (i.e., sensitivity and specificity are below 100.0%) [29].

## Methods

### Ethics statement

The Department of Parasitology-Mycology-Tropical Medicine (DPMTM) of the Faculty of Medicine of the Université des Sciences de la Santé is a reference laboratory for parasitic diseases diagnosis in Gabon. The study was performed in collaboration with the Ministry of Health which is the regulatory organ represented by the Parasitic Diseases National Control Programme (Programme National de Lutte contre les Maladies Parasitaires) andapproved by the National Ethical Committee for the Research in Gabon (PROT N°0081/2019/PR/SG/CNER). This study was nested in the routine procedures and quality control of readings at DPMTM, and no supplementary blood collection was performed. Each patient was informed about the current study during their consultation and gave their oral consent to participate.

### Study site and population

The study was conducted in the town of Owendo at the parasitology laboratory of the DPMTM of the Faculty of Medicine, Université des Sciences de la Santé of Gabon, between July 2018 and July 2019.

Once a week, the DPMTM receives patients for specialized consultations. Patients were enrolled in the study based on the presence of symptoms (see the “Definitions” section) and/or if they had required parasitological testing. Each patient has a medical folder stored in the archive. Among patients consulted in the DPMTM, the prevalence of diagnosed loiasis mono- and co-infection was approximately 8.2–64.0% [1, 30, 31]. Populations who undergo consultation are originally from the nine provinces of Gabon [1].

The current project was planned as a cross-sectional study. On the consultation days, the study team approached all patients to inform them about the availability of the study and to receive their verbal approval to use their blood sample for the specific research objective. For patients who consented, their blood samples were then used for the direct blood examination, leukoconcentration technique, and one supplementary test, the TBS-50.

### Study design

Venous blood samples (5 ml) were collected in ethylenediaminetetraacetic acid (EDTA) tubes between 10:00 and 14:00 on the same day in dedicated rooms. A thin blood smear was obtained for the determination of eosinophilia. Then, samples were transferred to the parasitological laboratory of the DPMTM to perform direct blood examination of 10 μl samples and TBS-50 using a calibrated micropipette. Finally, leukoconcentration analysis was performed.

The latter represents the reference test in the present study. This technique is supposed to have a higher sensitivity (100.0%) than the direct examination of total blood in participants with low parasitic load [1, 28]. These performance indicators were calculated from data extracted from epidemiological studies for the purpose of the current study. These epidemiological studies were not designed for the evaluation of the diagnostic tests. All data were recorded from the laboratory registries and the patients’ medical folders.

### Loiasis diagnosis

#### Direct blood examination

Using a calibrated micropipette, 10 μl of uncoagulated fresh whole blood was analyzed on a clean microscope slide using a coverslip at magnification of ×10 for spotting and ×40 for the microfilarial species identification. The entire preparation was read, and microfilaremia was determined according to the following formula:

$${\text{Microfilaremia}} = 100 \times {\text{number of counted microfilariae}}$$

#### Thick blood smear of 50 μl

Fifty microliters of fresh venous blood collected in an EDTA tube was spread out on a microscope slide (30 mm $\times$ 20 mm; (600 mm^2^) using a micropipette. The preparation was then dried at laboratory temperature for 24 h. Two milliliters of distilled water was added to cover the TBS for 5 min for the dehemoglobinization process. The dried slide was fixed with absolute methanol (100%) for 1 min and then dried again at laboratory temperature before staining with 2 ml of Giemsa 10% for 20 min. The staining solution was discarded, and the slide was rinsed with clean water to remove the excess Giemsa solution (Additional file 1: Fig. S1). The whole preparation was read, and microfilaremia was determined according to the following formula:

$${\text{Microfilaremia}} = 1000 \times \frac{{{\text{Number}}\;{\text{of}}\;{\text{microfilariae}}\;{\text{counted}}}}{{50\;{\text{microliters}}}}$$

#### Leukoconcentration

The third technique assessed in this study was leukoconcentration, which requires 4–5 ml of blood and was described by Ho and Petithory [13]. Briefly, a blood pellet was obtained after centrifugation at 1500 rpm for 3 min. Then, 2 ml of NaCl 9‰ and 1 ml of saponin 2% were added successively. Ten minutes later, the homogenate and the pellet obtained after centrifugation were processed under the same conditions (1500 rpm for 3 min). Microfilaremia was determined as follows:

$${\text{Microfilaremia}} = \frac{{{\text{Number}}\;{\text{of}}\;{\text{microfilariae}}\;{\text{counted}}}}{{{\text{five}}\;{\text{milliters}}}}$$

#### Interpretation of results

The preparation was considered negative if no *L. loa* microfilariae were found and positive in the presence of at least one.

### Eosinophil count

From a thin blood smear of 5 μl, eosinophils were counted and expressed as a percentage of 100 white blood cells detected under a light microscope. A normal rate of eosinophils was between 1 and 6%. An eosinophil proportion of 7% or more was considered hypereosinophilia.

### Quality control

The microscopists were granted access to the clinical information of each patient before reading the slides. All slides were labeled before performing parasitological analysis. Direct blood examinations and thick smears were carried out at the same time. Then, EDTA tubes were used for the leukoconcentration technique within 5 min. Slides were cleaned with lint-free wipes before use. Two expert microscopists read all slides independently. If there were discrepancies in the species identification, microfilariae detection (positive/negative result), or microfilaremia determination (a high microfilaremia/low microfilaremia ratio of less than 1.5), a third read was provided by an independent microscopist. Positive/negative results were confirmed from two concordant results from three microscopists. For microfilaremia, the mean was calculated from the closest results.

### Sample size calculation

The expected prevalence of microfilaremic loiasis in patients in the hospital environment is 8.2% based on a study by M’Bondoukwé et al. [31]. Sample size calculations were performed using nQuery 9 (version 9.2.1.0). For a sample size of 181, a two-sided 95% confidence interval for a single proportion using the large sample normal approximation would extend 0.04 from the observed proportion for an expected proportion of 8.2% [7.8–8.6].

### Definitions

Age was categorized into two age groups: 15–64 years, which is the high-risk age category, and other age groups, which included both patients younger than 15 years and older than 64 years [32]. Among patients with symptoms, we defined three groups: loiasis-related symptoms, other symptoms not specific to loiasis, and patients with loiasis-related symptoms + other symptoms. Calabar swelling, eye worm, and sensation of crawling under the skin were considered loiasis-related symptoms. Apparent prevalence was defined as the number of positive cases out of the total population obtained after direct examination of 10 μl blood, and true prevalence was defined as the number of positive cases out of the total population after leukoconcentration of 5 ml.

### Statistical analysis

Data were processed using Microsoft Excel 2016 with an operator and a verifier. Missing data were only observed for the eosinophil count. All the other parameters were filled for included patients. Categorical variables were summarized in absolute numbers and percentages. The normality of distribution of quantitative variables was assessed using the Shapiro–Wilk test. For quantitative data, age followed a normal distribution and was expressed using the mean (± standard deviation), whereas the percentage of eosinophils was presented using the median (first quartile–third quartile), as the normality assumption was not satisfied. Microfilaremia was expressed using the geometric mean [minimum–maximum] and compared between diagnostic tests using the nonparametric Wilcoxon test. Missing data were not imputed.

The leukoconcentration method was used as the reference gold standard according to previous results showing better sensitivity and specificity for this technique in the DPMTM relative to the direct blood examination [1]. Assuming it was a perfect test, the sensitivity and specificity of the TSB-50 and the direct examination were calculated using the classical formula (Table 1). The estimated performance parameters for diagnostic criteria (sensitivity, specificity, NPV, positive predictive value [PPV]) were calculated with their 95% exact confidence intervals (95% CI) using the *epiR* package.

**Table 1:** Contingency table and formulas for diagnostic accuracy

	Leukoconcentration positive	Leukoconcentration negative
Positive thick smear/direct examination	True positive (TP)	False positive (FP)
Negative thick smear/direct examination	False negative (FN)	True negative (TN)

Calculations for sensitivity (Se) = TP/(TP + FN), specificity (Sp) = TN/(TN + FP), positive predictive value (PPV) = TP/(TP + FP), and negative predictive value (NPV) = TN/(TN + FN) correspond to classical formulas for the determination of performance indicators

The area under the curve (AUC) was also computed. Exploratory descriptive analyses were performed, and test accuracy was stratified by age, sex, symptoms, and eosinophilia. Statistical analyses were performed with R software (version 4.2.2) using the *epiR* [33] and *pROC* [34] packages.

Next, we assumed that the leukoconcentration technique was an imperfect test, as it was found to yield false-negative results in comparison with a molecular diagnostic tool [35]. To evaluate the diagnostic test accuracy in the presence of an imperfect gold standard, we used the BLCM. Two tests were considered in each model: leukoconcentration and TBS50/direct blood examination (equivalent performance was assumed due to perfectly concordant results).

Three Bayesian models differing in their parameter prior distributions were fitted.

BLCM model I: This model utilizes information from the literature to derive prior distributions of the parameters. For the initial prevalence of the disease, we used the information from a published study performed at the DPMTM [31]. Initial sensitivity, specificity, PPV, and NPV were estimated using the Noireau and Apembe publication for the TBS-50 technique and the direct blood examination [28]. For the leukoconcentration technique, initial diagnostic accuracy values were obtained from a study performed in the same institution and laboratory [36]. For the calculation of priors based on these initial estimates, the *findbeta* function from the *PriorGen* package was employed [37].

Alternative model II: Uninformative priors were chosen for the parameters of the two tests (*sensitivity, specificity, NPV, PPV*) and the prevalence of the disease, corresponding to beta(1,1) prior distributions, assuming there were no relevant estimates available in the literature.

Alternative model III: The initial prevalence of the disease was taken from the study performed at DPMTM [31], and a prior was derived based on the prevalence estimate. For the diagnostic accuracy of the two tests, beta(1,1) prior distributions were used.

The Markov chain Monte Carlo (MCMC) algorithm was run for two chains, 100,000 iterations each, with the burn-in parameter set to 5000, ensuring that the process of simulation was optimal for the model parameter estimation. The MCMC plots were investigated for convergence of the algorithm. The resulting estimates are presented as medians with their 95% credible intervals (95% CI). The model was fit in R software using the *run.jags* function from the *runjags* package [38].

The results of the Bayesian analysis were concluded based on BLCM model I, and the two additional models (alternative models II and III) were fit to investigate the behavior of estimates when uninformative priors on prevalence, sensitivity, specificity, PPV, and NPV were used as a form of sensitivity analysis.

The model specifications are summarized in Table 2. Exploratory BLCM was also performed in the subgroups by age, sex, symptoms, and eosinophilia.

**Table 2:** Prior distributions derived from previous literature for the disease prevalence and performance characteristics of the tests

Models	Prior distribution								
	Prevalence	Leukoconcentration				TBS-50/direct blood examination			
		Sensitivity	Specificity	PPV	NPV	Sensitivity	Specificity	PPV	NPV
I	Beta(4.61,48.21)	Beta(52.21,0.33)	Beta(202.31,30.25)	Beta(84.49,29.75)	Beta(122.37,0.33)	Beta(15.72,8.02)	Beta(63.65,0.33)	Beta(10.73,0.33)	Beta(80.46,6.36)
II	Beta(1,1)	Beta(1,1)T(1-sp[1])^a^	Beta(1,1)	Beta(1,1)	Beta(1,1)	Beta(1,1)T(1-sp[1])	Beta(1,1)	Beta(1,1)	Beta(1,1)
III	Beta(4.61,48.21)	Beta(1,1)T(1-sp[1])	Beta(1,1)	Beta(1,1)	Beta(1,1)	Beta(1,1)T(1-sp[1])	Beta(1,1)	Beta(1,1)	Beta(1,1)

*PPV* positive predictive value, *NPV* negative predictive value

^a^T(1-sp[1]) = technical term used in JAGS (Just Another Gibbs Sampler) software when defining the formula of the Hui-Walter BLCA model. This addition to the default formula is a safeguard that would have an effect in scenarios when JAGS estimates the Se/Sp to be 0%. This additional “T(1-sp[1])” component of the prior then forces JAGS to consider this Se/Sp as 100% instead, as 0% Se/Sp = 100% Se/Sp after simply switching the labeling of the test results. For the models used in this paper, inclusion/exclusion of “T(1-sp[1])” had no effect on the estimates of the test performance characteristics, meaning that this safeguard remained unused

To calculate priors for the thick smear test, data from Noireau and Apembe [28] were used. Se: 66.7% (49.8–80.9); Sp: 100.0% (97.7–100.0); PPV: 100.0% (86.8–100.0); NPV: 93.0% (87.6–96.0). For the leukoconcentration, data from Bouyou-Akotet et al. [36] were used: Se: 100% (97.2–100.0); Sp: 87.1% (83.2–90.5); PPV: 74.1% (67.0–80.5); NPV: 100.0% (98.8–100.0)

## Results

In total, 191 patients were recruited (Additional file 1: Fig. S2). Their baseline characteristics are summarized in Table 3. The mean age was 46.7 ± 16.6 years. More than three fourths of the study population was female, and the gender ratio was 0.37. Slightly more than half of the patients were symptomatic (52.9%, *n* = 101/191): generalized pruritus was the most frequent clinical sign (20.4%; *n* = 39/191), and eye worm was the least frequent symptom (3.1%; *n* = 6/191). The number of eosinophils was available for 187 patients, and 49.7% (*n* = 93/187) presented hypereosinophilia.

**Table 3:** Characteristics of the study population

	*N*	%
Gender	191	
Male	52	27.2
Female	139	72.8
Age groups	191	
15–64 years	157	82.2
Other age groups	34	17.8
Patients with symptoms	191	
Yes	101	52.9
No	90	47.1
Calabar swelling	191	
Yes	11	5.8
No	180	94.2
Eye worm	191	
Yes	6	3.1
No	185	96.9
Sensation of crawling under the skin	191	
Yes	14	7.3
No	177	92.7
Generalized pruritus	191	
Yes	39	20.4
No	152	79.6
Other edema	191	
Yes	27	14.1
No	164	85.9
Other symptoms	191	
Yes	55	28.8
No	136	71.2
Hypereosinophilia	187^a^	
Yes	93	49.7
No	94	50.3

	Mean	Standard deviation
Age in years	46.7	16.6

	Median	First quartile–third quartile
Eosinophilia in %	6.0	2.5–17.5

^a^Four thin blood smear slides were not available for the eosinophil count

### Prevalence of microfilaremic loiasis assuming perfect test and density of *Loa loa* microfilaria

**Fig. 1:**
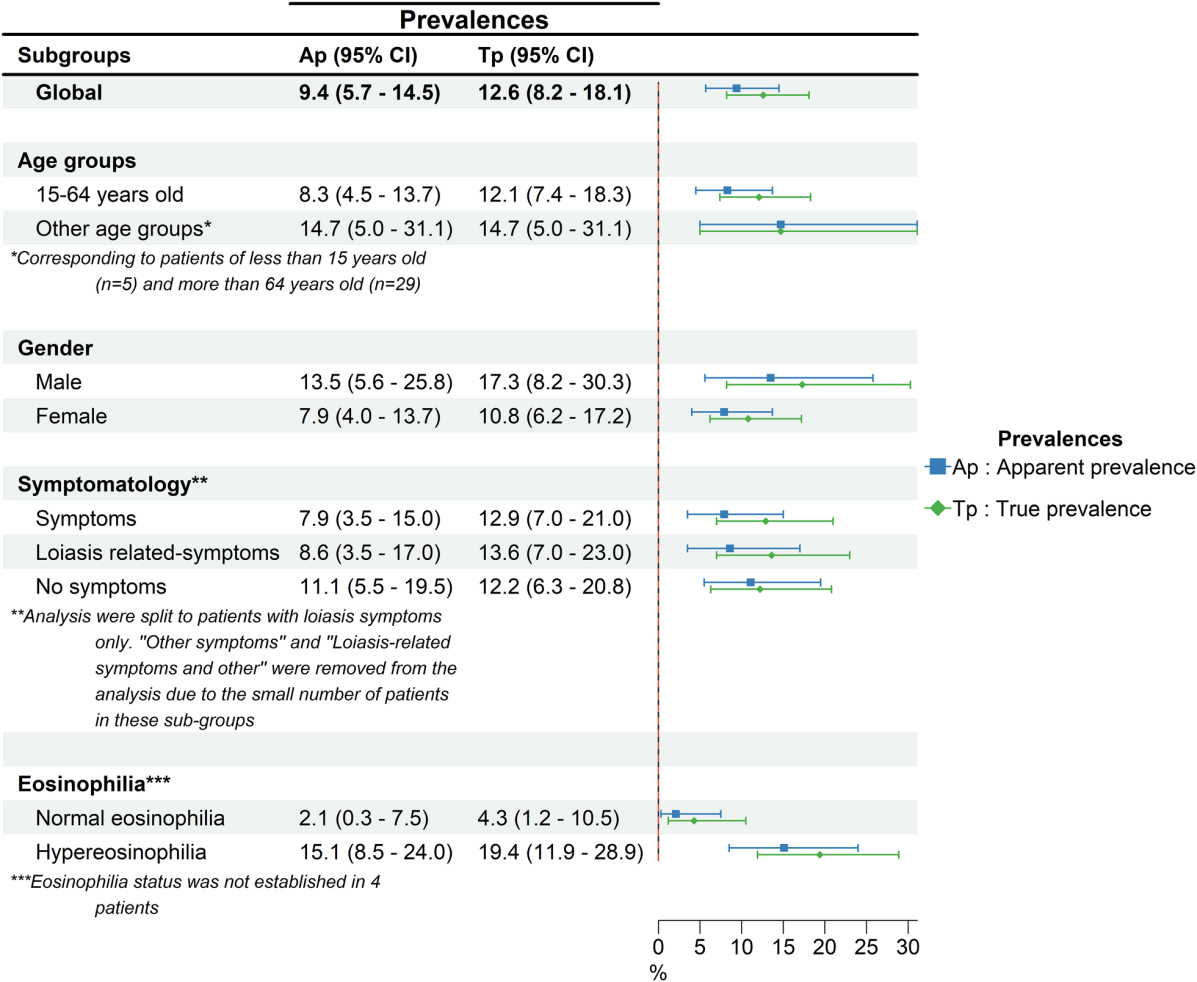
Prevalence of loiasis according to the direct blood examination, thick blood smear of 50 µl (Ap), and leukoconcentration technique (Tp). TBS-50 and direct blood examination yielded the same prevalence values

Considering the results of direct blood examination and TBS-50 in 191 patients, the prevalence of loiasis (apparent prevalence) was 9.4% [5.7–14.5] (*n* = 18/191). The microfilaremia geometric mean was 1006 [±5] mf/ml for the direct examination technique. Out of the 18 positive patients, three patients did not have a result for microfilaremia with TBS-50 due to the absence of biological material after Giemsa staining. The microfilaremia geometric mean was 816 (±5) mf/ml for the remaining 15 patients. The comparison of microfilaremia obtained with the direct blood examination and TBS-50 showed no significant difference (Wilcoxon signed-rank test, *Z* = −0.031, *P* = 0.97). After applying the leukoconcentration technique, six patients out of 173 who tested negative with the direct blood examination were positive, and the global prevalence (true prevalence) was 12.6% [8.2–18.1] (*n* = 24/191). For these six negative slides at the direct examination/TBS-50 and positive results with the leukoconcentration technique, a mean of three microfilaremia were detected (±2) mf/ml. Prevalence and absolute values are summarized in Fig. 1 and Additional file 1: Fig. S2.

Stratified exploratory analyses are summarized in Fig. 1. In the case of hypereosinophilia, the prevalence determined by direct examination/TBS-50 and that by leukoconcentration were 7.2-fold (apparent prevalence) and 4.5-fold (true prevalence) higher than the values in the group of patients with normal eosinophilia.

### Determination of diagnostic performance using the classical formula

**Fig. 2:**
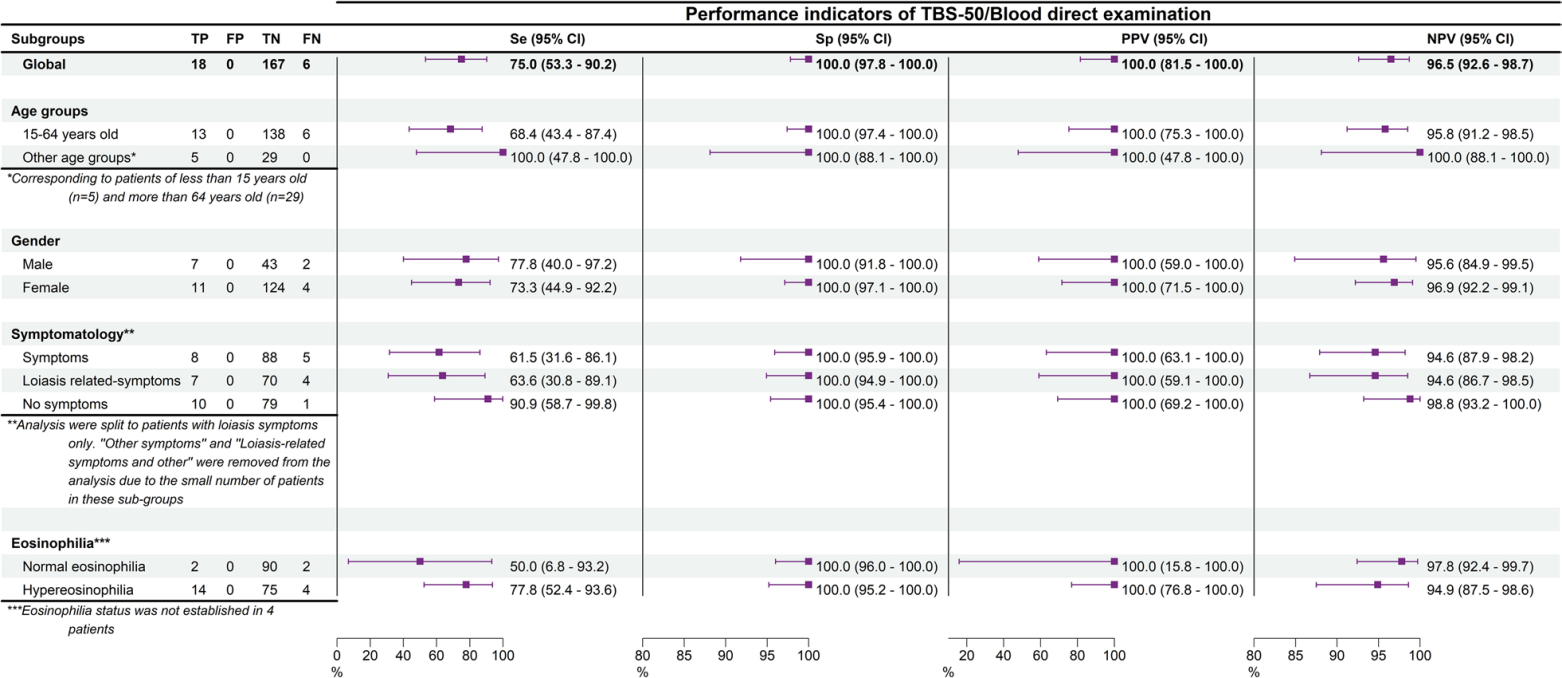
Diagnostic accuracy of TBS-50/direct blood examination globally and stratified by age, sex, symptomatology, and eosinophilia*.* TP: true positive; FP: false positive; TN: true negative; FN: false negative; Se: sensitivity; Sp: specificity; PPV: predictive positive value; NPV: negative predictive value. These results correspond to the “classical formula” using leukoconcentration as the perfect test. Columns 2 to 5 are numbers of observations

Considering leukoconcentration analysis as the perfect gold standard test, direct examination and TBS-50 presented the same diagnostic accuracy, as summarized in Table 4. The sensitivity, specificity, PPV, and NPV were 75.0% [53.3–90.2], 100.0% [97.8–100.0], 100.0% [81.5–100.0], and 96.5% [92.6–98.7], respectively (Table 4). Figure 2 displays the stratified analyses.

**Table 4:** Contingency table with the direct examination (10 µl) and thick blood smear (50 µl) versus leukoconcentration technique as the gold standard (reference technique)

	Leukoconcentration		Total
	Positive	Negative	
Direct examination			
Positive	18	0	18
Negative	6	167	173
Total	24	167	191
Thick blood smear of 50 µl			
Positive	18	0	18
Negative	6	167	173
Total	24	167	191

Considering direct examination of 10 ml of blood as a perfect reference test in comparison with the TBS-50, there were no discordant results between the two techniques (Table 5). Consequently, all performance indicator estimates were equal to 100.0%.

**Table 5:** Contingency table for thick blood smear (50 µl) versus the direct examination (10 µl) as the gold standard (reference technique)

	Direct examination		Total
	Positive	Negative	
Thick blood smear of 50 µl			
Positive	18	0	18
Negative	0	173	173
Total	18	173	191

Regarding the AUCs of direct blood examination and TBS-50 techniques, they were greater than 95.0% in all analyses performed globally and in the subgroup analyses by age, gender, symptoms, and eosinophilia (Table 6).

**Table 6:** Areas under the curve (AUCs) of TBS-50/direct blood examination overall and in the age, gender, symptomatology, and eosinophilia subgroups

Subgroups	AUC (%)	95% CI
Global	98.3	96.9–99.6
Age groups		
15–64 years	97.9	96.3–99.5
Other age groups	100.0	100.0
Gender		
Male	98.4	94.7–100.0
Female	97.8	96.9–99.9
Symptomatology		
Symptoms	97.3	95.0–99.6
No symptoms	99.4	98.1–100.0
Eosinophilia		
Normal eosinophilia	98.9	97.4–100.0
Hypereosinophilia	97.5	95.0–99.9

### Determination of diagnostic performances using Bayesian latent class analysis

In the BLCM model I including informative priors on prevalence and diagnostic accuracy, the overall prevalence of microfilaremic loiasis was 9.7% [6.2–13.7] based on either TBS-50 or leukoconcentration. Leukoconcentration demonstrated nearly perfect sensitivity of 99.9% [97.8–100.0] and specificity of 91.4% [88.6–94.1]. The PPV and NPV were 74.1% [66.0–81.7] and 99.9% [98.8–100.0], respectively. With regard to TBS-50 and direct examination, the sensitivity was 78.9% [65.3–90.3] and the specificity was 100.0% [99.3–100.0]. NPV and PPV were very high (above 90.0%), similar to the results obtained via the classical formula (assuming that leukoconcentration is the reference test).

The main results and exploratory results, stratified by age, sex, symptomatology, and eosinophilia, are presented in Table 7. The sensitivity of the TBS-50 and direct examination was lowest in the subgroup of patients with normal eosinophilia, similar to the classical analysis.

**Table 7:** Diagnostic accuracy using Bayesian latent class analysis: BLCM model I

Model I	Subgroups	Median (95% credible interval) (%)									
Parasitological technique		Prevalence		Sensitivity		Specificity		Positive predictive value		Negative predictive value	
Direct blood examination (10 µl)/thick blood smear (50 µl)	Global	9.7	6.2–13.7	78.9	65.3–90.3	100.0	99.3–100.0	99.1	87.2–100.0	93.0	87.3–97.7
	Age group										
	15–64 years	8.8	5.1–13.0	76.3	62.4–90.0	99.9	99.2–100.0	99.1	86.7–100.0	93.0	87.3–97.7
	Other age groups^b^										
	Gender										
	Male	11.3	5.5–17.7	73.3	56.2–87.3	99.9	98.6–100.0	99.1	86.8–100.0	93.0	87.3–97.6
	Female	8.4	4.6–12.6	75.7	61.0–88.9	99.9	99.1–100.0	99.1	86.9–100.0	93.0	87.2–97.6
	Symptomatology										
	Symptoms^b^										
	Loiasis-related symptoms	9.1	4.6–14.6	72.3	56.4–87.5	99.9	98.9–100.0	99.1	86.5–100.0	93.0	87.2–97.6
	No symptoms	10.2	5.7–15.6	76.1	61.2–89.3	99.9	98.9–100.0	99.1	87.3–100.0	93.0	87.2–97.7
	Eosinophilia										
	Normal	4.5	1.5–8.2	68.7	50.2–85.3	99.9	99.9–100.0	99.1	86.9–100.0	93.0	87.1–97.6
	Hypereosinophilia^b^										
Leukoconcentration(5 ml)^a^	Global	9.7	6.2–13.7	99.9	97.8–100.0	91.4	88.6–94.1	74.1	66.0–81.7	99.9	98.8–100.0
	Age groups										
	15–64 years	8.8	5.1–13.0	99.8	97.7–100.0	90.7	87.6–93.5	74.0	66.0–82.1	98.8	98.8–100.0
	Other age groups^b^										
	Gender										
	Male	11.3	5.5–17.7	99.8	97.5–100.0	88.6	84.9–92.3	74.1	65.4–81.7	99.9	98.8–100.0
	Female	8.4	4.6–12.6	99.8	97.5–100.0	90.8	87.6–93.6	74.1	66.0–81.9	99.9	98.8–100.0
	Symptomatology										
	Symptoms^b^										
	Loiasis-related symptoms	9.1	4.6–14.6	99.8	97.5–100.0	89.1	85.4–92.4	74.1	65.9–81.9	99.9	98.8–100.0
	No symptoms	10.2	5.7–15.6	99.8	97.4–100.0	90.1	86.7–93.3	74.1	66.1–82.2	99.9	98.8–100.0
	Eosinophilia										
	Normal	4.5	1.5–8.2	99.8	97.2–100.0	90.2	86.9–93.4	74.1	65.8–81.7	99.9	98.8–100.0
	Hypereosinophilia^b^										

^a^Imperfect reference test

^b^Model did not converge for this particular population subgroup

Sensitivity analysis models are presented in Additional file 1: Table S1 (model II) and Table S2 (model III). When using uninformative priors, the overall median prevalence of loiasis remained approximately 10%, with 11.2% [6.7–16.4] in alternative model II and 10.2% [6.3–14.4] in alternative model III. The median prevalence was the highest in patients with hypereosinophilia and the lowest in those with normal eosinophilia. In both sensitivity analysis models, the overall median sensitivity and specificity for leukoconcentration were high, exceeding 95%. Compared to BLCM model I, TBS-50 and direct examination had higher median sensitivity when using uninformative priors, estimated as 84.2% [64.0–100.0] for diagnostic accuracy and prevalence, and as 86.3% [65.5–100.0] for diagnostic accuracy only. In all models, TBS-50 and direct examination had nearly perfect specificity. In contrast to BLCM model I, PPV and NPV were only approximately 50.0% in the alternative models for all three diagnostic tools. However, the confidence intervals were extremely wide, ranging from 0 to 100% for NPV and PPV. While specificity was similar across subgroups, the median sensitivity of any diagnostic test was the lowest in the subgroup of patients with normal eosinophilia, similar to the results obtained in BLCM model I.

## Discussion

In co-endemic areas, loiasis is a major obstacle in the control of onchocerciasis, a parasitic NTD. Indeed, the occurrence of SAEs has been recorded after IVM or DEC administration in populations with high *L. loa* microfilaremia. Moreover, recent studies have shown an increased risk of mortality in individuals with filaremia greater than 30,000 mf/ml, highlighting its importance in public health research [10, 39]. During a national campaign for loiasis cartography in Gabon, TBS-50 was used as the microfilaremic loiasis diagnostic. However, to the best of our knowledge, the performance characteristics of this technique were not clearly described in the literature. Similarly, there is little information regarding direct examination of 10 µl blood samples or leukoconcentration analysis of 5 ml of blood—two other commonly used diagnostic techniques. The present study aims to fill this research gap using the Standards for the Reporting of Diagnostic Accuracy Studies–Bayesian Latent Class Model (STARD-BLCM) criteria (Additional file 2: Table S3).

In our study, the percentage of carriers of *L. loa* microfilariae identified with the leukoconcentration technique was 12.6% [8.2–18.1] in the classical analysis and 9.7% [6.2–13.7] in the BLCM. Previous studies performed in the same laboratory with an identical diagnostic tool found prevalence of 8.2% and 39.5% [1, 31]. The place of residence of the populations in these studies may partially explain the differences in prevalence observed. Indeed, people who live in endemic areas, mainly in rural settlements, are more exposed to *Chrysops* (loiasis vector) bites than their counterparts living in urban areas [4, 31, 40]. In the survey performed by Bouyou-Akotet et al., for example, two thirds of the population came from rural areas, which can explain the higher prevalence observed in that study [1]. Microfilaremia levels identified via direct blood examination were slightly higher than those obtained via the TBS-50 technique, but the differences were not statistically significant, with a *P*-value of 0.97. Direct blood examination is performed with the fresh blood of patients and allows observation of living *L. loa* microfilarial mobility. On the other hand, the microfilariae can cross the slide and may be counted more than once, in contrast to TBS-50, which fixes worms on the preparation. In the current study, three TBS-50 slides could only be partially analyzed, as the biological material was removed during the staining process. This may happen with venous blood collection in anticoagulant tubes, contrary to capillary collection, where better adherence of the blood to the slide is observed. In addition, a recent study conducted in Lambaréné and surrounding villages in Gabon found that the odds of microfilaremia detection were 1.24 higher from capillary blood than from venous blood [41]. This may influence the diagnostic accuracy of the TBS-50 and could be considered one of the limitations of the evaluation of the TBS method in the current survey.

In our study, we first assessed the performance of TBS-50 and direct examination compared to leukoconcentration, assuming the latter is a perfect reference technique. Although TBS-50 requires five times as much blood as direct blood examination, there were no discordant results between the two techniques. It follows that, using leukoconcentration as a perfect reference technique, the diagnostic accuracy of the TBS-50 and direct blood examination was identical, with sensitivity of 75.0% [53.3–90.2] and specificity of 100.0% [97.8–100.0]. The PPV was 100.0% [81.5–100.0] and the NPV was 96.5% [92.6–98.7]. In the literature, only one other study had a similar design [28]. In this study, the researchers used two slides of 20 µl each (40 µl in total) in comparison with saponin hemolysis, which requires a volume of 5 ml of blood, similar to the leukoconcentration technique [28]. Although the specificity and the PPV were the same as in our study (100.0%), the sensitivity and NPV were slightly lower in that study (67.0% and 93.0%, respectively). It is possible that the additional collection of 10 µl for TBS-50 in the current study led to increased sensitivity and NPV. On the other hand, it is well known that high prevalence is associated with a lower NPV [42]. The prevalence found by Apembe and Noireau was 1.5- and twofold higher than that in our study (19.4% versus 12.6/9.7%) [28].

A perfect diagnostic test can ideally discriminate subjects with and without a disease. However, such tests with performance characteristics at 100.0% do not exist [43]. With blood volume less than 5 ml in a study performed in Franceville, Gabon, the leukoconcentration technique showed false-negative results in *L. loa* microfilaremic individuals when compared with the results of a nested PCR assay [35]. Another study showed a difference of almost 50.0% between a concentration technique and PCR for the detection of cases [44]. This imperfection translates into biased estimates when using the classical formula. To overcome this limitation, we applied a BLCA approach that can be used in the presence of imperfect reference tests [45–47]. This statistical method has been increasingly used in recent years; however, it has not yet been applied in Gabon in the context of diagnostic testing. Bayesian analysis allows us to estimate diagnostic performance characteristics of the reference imperfect test and the index test, as well as to estimate the proportion of *L. loa* microfilariae carriers [48, 49].

In the BLCM model I including prior information from the literature, the BLCM analysis showed higher, nearly perfect sensitivity for the leukoconcentration technique compared to TBS-50 and direct blood examination. However, leukoconcentration had a false-positive rate of 8.6% (= 100.0–91.4 for specificity). This is a low but nonzero rate, with less than 1 in 10 cases that are falsely declared positive. Indeed, the preparation observation in monochrome white color settings could easily lead to confusion of immobile microfilariae with nonparasitic elements. A potential option to reduce the rate of false-positive results is the addition of a staining step to the leucocyte pellet. This will decrease errors by microscopists. However, in the literature, the possibility of an additional staining step was not mentioned. In Congo settings, the observed rate of false positives was higher (33.3%) with two TBS-20 [28].

Regarding the TBS-50 and direct blood examination, there was a 21.1% (= 100–78.9% for the sensitivity estimate) rate of false-negative results—that is, more than one fifth of the screened population who had a negative test result had the disease. This is a high rate and could be considered the main limitation of these diagnostic tests. Nevertheless, the other accuracy parameters are all above 90.0%, with perfect specificity of 100%.

To assess the robustness of our results, we conducted sensitivity analyses using uninformative priors for prevalence and test accuracy. The results were similar when only priors for test accuracy were assumed to be uninformative or the prevalence prior. At first sight, it seems that the prior associated with the prevalence does not influence the model. However, we could not confirm this hypothesis because when removing the prior associated with the prevalence while keeping those associated with the TBS-50 and leukoconcentration, the model did not converge due to incompatibility between the priors and the data. Sensitivity estimates increased for TBS-50 and direct examination when using uninformative priors (alternative models) compared to the scenario when informative priors were used (BLCM model I). However, the results comparison should be conducted with caution, as credible intervals largely overlap between all three models. It was observed that with the use of uninformative priors, the credible intervals for NPV and PPV were very wide (with the point estimates at approximately 50%), reflecting high uncertainty in the estimates of NPVs and PPVs, which was not the case in the model with all priors being informative. One of the reasons for this is that the absence of prior information obviously decreases the ability of the model to precisely estimate all of the diagnostic characteristics, especially taking into account the relatively small sample size of the study itself. On the other hand, certainty in all of the estimates achieved in BLCM model I, where all priors were informative, was also expected, as the more informative the priors are, the more certain are the estimates drawn from them.

In the current study, we also observed that sensitivity was higher in a subgroup with hypereosinophilia and lower in those with normal eosinophilia, regardless of the diagnostic test considered. Eosinophils are involved in the immune response against helminth pathogens. In low-income and developing countries of Central and West Africa, eosinophilia is often found to be significantly associated with helminth infection and with loiasis, such as in Lambaréné in Gabon [50]. Eosinophils secrete immunoglobulin E, which is implicated in allergic reaction: a positive correlation was observed during *L. loa* infection [30]. Eosinophils can potentially be a good marker for microfilaremic loiasis in Gabon in high-prevalence settings. However, *M. perstans* and intestinal parasites were not investigated in the study population, which limited the development of a real picture of loiasis and eosinophil production. They also influence the human immune system toward the regulatory T cell/T helper 2 cell (Treg/Th2) polarized response [30, 51].

The deployment of a test depends on both the sensitivity and specificity. The TBS-50 is used in Gabon in mass surveys but generally not for diagnostic testing. One of the qualities recommended for a screening test when a trade-off has to be made is good sensitivity at the expense of specificity. TBS-50 has sensitivity of approximately 80.0% and is currently the best tool that can be easily implemented with limited resources. In addition to the previously mentioned criteria, a screening test should be well accepted by the populations [52]. In our study, TBS-50 was not tested in communities but rather in populations coming for medical consultations in the hospital environment. Future research should investigate the acceptability of the TBS-50 among the general population in Gabon.

## Conclusions

In the present study conducted at DPMTM, we evaluated the performance characteristics of three loiasis techniques that are regularly used in Gabon, namely, leukoconcentration, TBS-50, and direct blood examination. We performed BLCA that adjusts for the imperfect accuracy of this test method. The median prevalence of microfilaremic loiasis was approximately 10.0%. Whereas specificity estimates were comparable for the three tools (> 90%), the estimated sensitivity of the leukoconcentration technique was superior to that of TBS and direct blood examination, with estimates of approximately 80% for the latter compared to nearly 100% for leukoconcentration. The TBS-50 technique can be used for mass surveys by national parasitic disease control programs and is easily deployed in rural areas. However, one should be aware that a false-negative result will be obtained in one of five cases. As these patients generally have low microfilaremia levels, they are not at risk for SAEs during mass drug administration with IVM. However, as loiasis will not be diagnosed in those patients, they will not be managed for their disease, aside from administration of IVM.

### Supplementary Information

**Additional file 1: Figure S1.** Realization of the thick blood smear of 50 µl. **Figure S2.** Flow diagram of the study. **Table S1.** Diagnostic accuracy using the Bayesian latent class analysis: Alternative model II. **Table S2.** Diagnostic accuracy using the Bayesian latent class analysis: Alternative model III.

**Additional file 2: Table S3.** Criteria of a study conducted according to the Standards for the Reporting of Diagnostic Accuracy Studies that use Bayesian latent class models.

## Data Availability

The datasets used during the current study are available as supplementary materials.

## Abbreviations

AUC: Area under the curve
BLCM: Bayesian latent class model
DEC: Diethylcarbamazine
DPMTM: Department of Parasitology-Mycology-Tropical Medicine
EDTA: Ethylenediaminetetraacetic acid
IVM: Ivermectin
LAMP: Loop-mediated isothermal amplification
*L. loa*: *Loa loa*
MCMC: Markov chain Monte Carlo
NPV: Negative predictive value
NTD: Neglected tropical disease
PCR: Polymerase chain reaction
PPV: Positive predictive value
SAEs: Severe adverse events
TBS-50: Thick blood smear of 50 µl
Th2: T helper 2
