# Supplementary material for: Low diagnostic performance of thick blood smears of 50 µl in comparison with direct examination of 10 µl blood and the leukoconcentration technique of 5ml blood among loiasis-suspected patients with low microfilaremia in Gabon, Central Africa, using the STARD-BLCM guidelines
Source: Parasit Vectors. 2024 Mar 15;17:138. doi: 10.1186/s13071-023-06089-1 (PMC10943916; doi:10.1186/s13071-023-06089-1)
Supplement: Supplementary file 1 — Additional file 1: Figure S1. Realization of the thick blood smear of 50 µl. Figure S2. Flow diagram of the study. Table S1. Diagnostic accuracy using the Bayesian latent class analysis: Alternative model II. Table S2. Diagnostic accuracy using the Bayesian latent class analysis: Alternative model III. [file 13071_2023_6089_MOESM1_ESM.docx]

**Additional file 1**

**Figure S1:** Realization of the thick blood smear of 50 µL

**
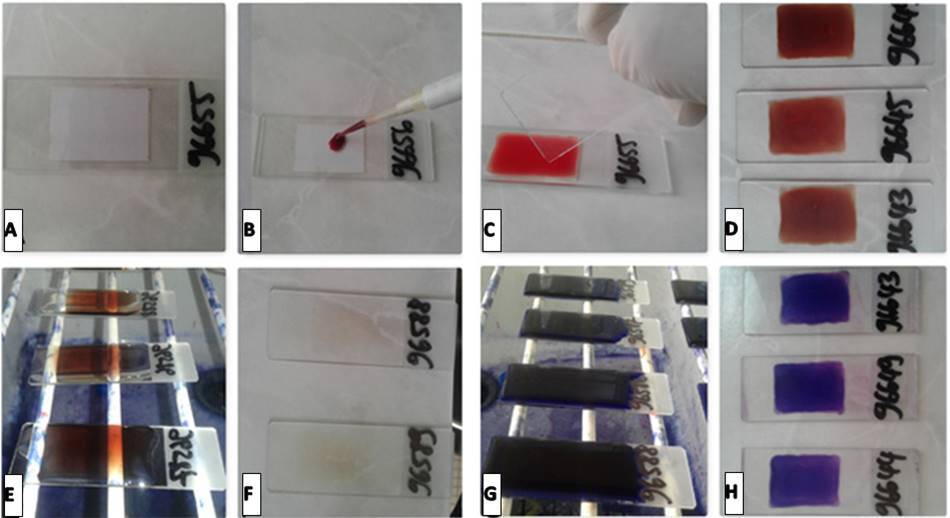
**

**A**: Identified microscope slide on a template of 30 mm $\times$ 20 mm. **B**: Depositing of a 50 µL blood drop using a calibrated micropipette. **C**: Spread of the blood on the microscope slide following the template. **D**: Dried thick blood smear after 24 hours. **E** and **F**: Dehemoglobinization with distilled water. **G**: Staining with Giemsa at 10% after fixation with absolute methanol. **H**: Thick blood smear ready to be read.

**Figure S2.** Flow diagram of the study

**
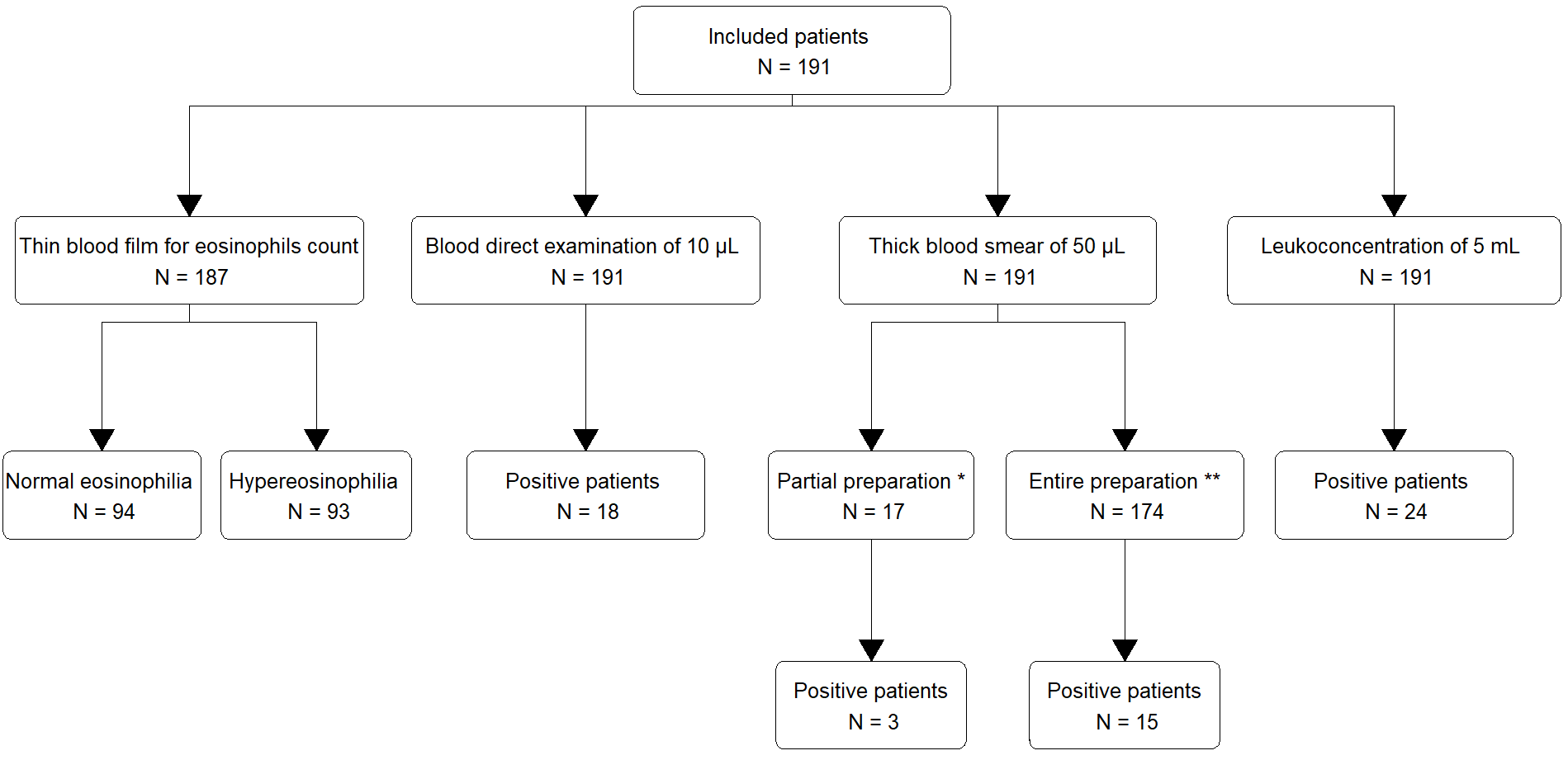
**

*During the staining process of the TBS-50, it is possible that the preparation can be removed from the slide. These slides were defined as “Partial preparation”. They were considered for the positivity of the slide (positive/negative) but not for the calculation of the microfilaremia. ****** The majority of the slides kept their total integrity and were defined as “Entire preparation”. From these slides, positivity and microfilaremia could be determined.

**Table S1.** Diagnostic accuracy using the Bayesian latent class analysis: Alternative model II

| Alternative model II | Subgroups | Median (95% Credible Interval) (%) | | | | | | | | | | |
| --- | --- | --- | --- | --- | --- | --- | --- | --- | --- | --- | --- | --- |
| Parasitological techniques |  | Prevalence | | Sensitivity | | | Specificity | | Positive Predictive Value | | Negative Predictive Value | |
| Blood direct examination of 10 microliters/  Thick blood smear of 50 microliters | Global | 11.2 | 6.7-16.4 | 84.2 | 64.0-100.0 | 99.6 | | 98.2-100.0 | 50.3 | 4.4-99.3 | 50.4 | 3.9-98.9 |
|  | Age groups |  |  |  |  |  | |  |  |  |  |  |
|  | 15-64 years old | 10.5 | 5.5-16.4 | 79.8 | 55.6-99.9 | 99.5 | | 97.8-100.0 | 50.9 | 5.2-99.9 | 50.2 | 1.5-96.2 |
|  | Others age groups | 16.4 | 5.6-29.8 | 87.2 | 55.4-100.0 | 97.5 | | 89.5-100.0 | 49.6 | 3.2-98.2 | 50.1 | 4.6-99.3 |
|  | Gender |  |  |  |  |  | |  |  |  |  |  |
|  | Male | 16.5 | 7.1-28.1 | 81.4 | 51.4-100.0 | 98.3 | | 92.9-100.0 | 50.0 | 0.3-95.3 | 50.1 | 0.6-95.4 |
|  | Female | 9.7 | 4.9-15.6 | 81.7 | 56.5-100.0 | 99.4 | | 97.6-100.0 | 49.9 | 5.2-100.0 | 50.2 | 1.3-96.2 |
|  | Symptomatology |  |  |  |  |  | |  |  |  |  |  |
|  | Symptoms | 9.9 | 4.3-16.6 | 83.5 | 55.5-100.0 | 99.2 | | 96.6-100.0 | 50.1 | 2.7-97.6 | 50.2 | 4.0-98.7 |
|  | Loiasis-related symptoms | 11.8 | 4.7-20.6 | 74.6 | 43.7-100.0 | 99.0 | | 95.7-100.0 | 50.0 | 2.4-97.4 | 50.1 | 5.1-100.0 |
|  | No symptoms | 12.3 | 6.0-19.5 | 89.7 | 67.4-100.0 | 99.1 | | 96.2-100.0 | 49.6 | 5.0-100.0 | 49.8 | 0.4-95.2 |
|  | Eosinophilia |  |  |  |  |  | |  |  |  |  |  |
|  | Normal | 4.1 | 0.2-10.8 | 58.0 | 16.7-100.0 | 99.2 | | 96.5-100.0 | 50.1 | 3.5-98.5 | 50.3 | 0.4-95.4 |
|  | Hypereosinophilia | 17.7 | 10.1-26.7 | 84.9 | 63.1-100.0 | 99.1 | | 96.0-100.0 | 49.3 | 0.6-95.6 | 50.3 | 0.0-95.1 |
| Leukoconcentration of 5 milliliters^1^ | Global | 11.2 | 6.7-16.4 | 96.2 | 84.8-100.0 | 97.8 | | 94.5-100.0 | 50.1 | 1.0-95.7 | 50.2 | 1.0-95.9 |
|  | Age groups |  |  |  |  |  | |  |  |  |  |  |
|  | 15-64 years old | 10.5 | 5.5-16.4 | 94.8 | 79.5-100.0 | 97.3 | | 93.4-100.0 | 49.8 | 1.2-95.9 | 49.7 | 5.0-99.7 |
|  | Others age groups | 16.4 | 5.6-29.8 | 87.1 | 54.5-100.0 | 97.5 | | 89.5-100.0 | 50.0 | 4.2-98.9 | 49.9 | 0.0-94.9 |
|  | Gender |  |  |  |  |  | |  |  |  |  |  |
|  | Male | 16.5 | 7.1-28.1 | 90.7 | 65.3-100.0 | 96.1 | | 88.2-100.0 | 50.3 | 0.1-94.9 | 49.6 | 0.4-95.2 |
|  | Female | 9.7 | 4.9-15.6 | 93.9 | 76.3-100.0 | 97.8 | | 94.2-100.0 | 50.0 | 1.9-96.8 | 49.4 | 0.6-95.1 |
|  | Symptomatology |  |  |  |  |  | |  |  |  |  |  |
|  | Symptoms | 9.9 | 4.3-16.6 | 91.9 | 69.0-100.0 | 98.1 | | 94.2-100.0 | 50.5 | 0.0-94.8 | 49.7 | 3.3-98.0 |
|  | Loiasis-related symptoms | 11.8 | 4.7-20.6 | 90.8 | 65.4-100.0 | 96.0 | | 89.7-100.0 | 50.0 | 0.9-95.6 | 49.4 | 4.0-98.6 |
|  | No symptoms | 12.3 | 6.0-19.5 | 93.3 | 74.6-100.0 | 98.5 | | 94.8-100.0 | 50.0 | 3.9-98.9 | 50.3 | 3.0-97.8 |
|  | Eosinophilia |  |  |  |  |  | |  |  |  |  |  |
|  | Normal | 4.1 | 0.2-10.8 | 73.8 | 27.1-100.0 | 97.9 | | 93.9-100.0 | 50.3 | 4.9-99.8 | 49.5 | 2.3-97.2 |
|  | Hypereosinophilia | 17.7 | 10.1-26.7 | 95.2 | 80.7-100.0 | 96.4 | | 90.6-100.0 | 50.1 | 1.4-96.4 | 49.9 | 0.8-95.4 |

**^1^**Imperfect reference test.

**Table S2.** Diagnostic accuracy using the Bayesian latent class analysis: Alternative model III

| Alternative model III | Subgroups | Median (95% Credible Interval) (%) | | | | | | | | | |
| --- | --- | --- | --- | --- | --- | --- | --- | --- | --- | --- | --- |
| Parasitological techniques |  | Prevalence | | Sensitivity | | Specificity | | Positive Predictive Value | | Negative Predictive Value | |
| Blood direct examination of 10 microliters/  Thick blood smear of 50 microliters | Global | 10.2 | 6.3-14.4 | 86.3 | 65.5-100.0 | 99.6 | 98.2-100.0 | 50.0 | 3.7-98.5 | 50.0 | 2.9-97.5 |
|  | Age groups |  |  |  |  |  |  |  |  |  |  |
|  | 15-64 years old | 9.4 | 5.3-10.6 | 82.3 | 57.5-100.0 | 99.5 | 97.8-100.0 | 50.3 | 1.0-95.8 | 49.6 | 3.9-98.7 |
|  | Others age groups | 10.9 | 5.0-18.0 | 87.7 | 58.2-100.0 | 97.4 | 89.1-100.0 | 49.4 | 4.7-99.7 | 49.8 | 1.4-96.5 |
|  | Gender |  |  |  |  |  |  |  |  |  |  |
|  | Male | 11.5 | 5.9-18.5 | 85.1 | 56.3-100.0 | 98.2 | 92.7-100.0 | 50.1 | 2.5-97.1 | 50.1 | 0.9-95.8 |
|  | Female | 8.9 | 4.8-13.4 | 83.2 | 58.3-100.0 | 99.4 | 97.5-100.0 | 50.2 | 2.8-97.6 | 49.6 | 0.3-95.1 |
|  | Symptomatology |  |  |  |  |  |  |  |  |  |  |
|  | Symptoms | 8.8 | 4.5-13.9 | 84.5 | 57.4-100.0 | 99.2 | 96.6-100.0 | 50.1 | 0.9-95.8 | 50.5 | 5.1-99.7 |
|  | Loiasis-related symptoms | 9.6 | 4.6-15.5 | 78.8 | 47.0-100.0 | 99.0 | 95.6-100.0 | 50.2 | 0.1-94.7 | 49.5 | 0.1-94.8 |
|  | No symptoms | 10.4 | 5.8-15.8 | 90.2 | 68.3-100.0 | 99.1 | 96.2-100.0 | 49.7 | 2.3-96.9 | 50.2 | 5.1-99.9 |
|  | Eosinophilia |  |  |  |  |  |  |  |  |  |  |
|  | Normal | 5.5 | 1.9-10.5 | 53.9 | 17.2-100.0 | 99.2 | 96.6-100.0 | 49.4 | 2.3-97.1 | 50.4 | 5.2-99.9 |
|  | Hypereosinophilia | 13.5 | 8.0-19.7 | 88.6 | 66.7-100.0 | 99.0 | 95.9-100.0 | 50.2 | 4.7-99.5 | 50.3 | 5.1-99.9 |
|  |  |  |  |  |  |  |  |  |  |  |  |
| Leukoconcentration of 5 milliliters^1^ | Global | 10.2 | 6.3-14.4 | 96.3 | 85.2-100.0 | 97.5 | 94.3-100.0 | 49.4 | 3.9-98.6 | 49.2 | 2.0-96.8 |
|  | Age groups |  |  |  |  |  |  |  |  |  |  |
|  | 15-64 years old | 9.4 | 5.3-10.6 | 95.0 | 80.0-100.0 | 96.9 | 93.1-100.0 | 50.4 | 3.4-98.3 | 50.5 | 2.1-96.8 |
|  | Others age groups | 10.9 | 5.0-18.0 | 87.6 | 57.5-100.0 | 97.4 | 89.1-100.0 | 49.9 | 0.0-94.8 | 50.0 | 1.1-95.7 |
|  | Gender |  |  |  |  |  |  |  |  |  |  |
|  | Male | 11.5 | 5.9-18.5 | 91.3 | 67.6-100.0 | 95.3 | 87.4-100.0 | 50.3 | 5.2-100.0 | 49.6 | 5.0-100.0 |
|  | Female | 8.9 | 4.8-13.4 | 94.1 | 77.0-100.0 | 97.6 | 94.1-100.0 | 50.8 | 4.9-99.8 | 49.7 | 2.7-97.2 |
|  | Symptomatology |  |  |  |  |  |  |  |  |  |  |
|  | Symptoms | 8.8 | 4.5-13.9 | 92.0 | 70.4-100.0 | 98.0 | 93.9-100.0 | 50.2 | 1.0-95.7 | 49.7 | 0.0-94.6 |
|  | Loiasis-related symptoms | 9.6 | 4.6-15.5 | 91.3 | 67.1-100.0 | 95.4 | 89.4-100.0 | 49.7 | 4.9-99.8 | 50.2 | 3.1-98.0 |
|  | No symptoms | 10.4 | 5.8-15.8 | 93.5 | 74.4-100.0 | 98.4 | 94.7-100.0 | 50.0 | 1.5-96.4 | 49.7 | 0.0-94.7 |
|  | Eosinophilia |  |  |  |  |  |  |  |  |  |  |
|  | Normal | 5.5 | 1.9-10.5 | 73.2 | 28.2-100.0 | 98.1 | 94.3-100.0 | 50.3 | 0.6-95.5 | 50.3 | 0.7-95.6 |
|  | Hypereosinophilia | 13.5 | 8.0-19.7 | 95.3 | 81.5-100.0 | 95.6 | 89.9-100.0 | 50.1 | 0.0-94.8 | 49.9 | 0.3-95.0 |

^1^Imperfect reference test
