# Supplementary material for: Low diagnostic performance of thick blood smears of 50 µl in comparison with direct examination of 10 µl blood and the leukoconcentration technique of 5ml blood among loiasis-suspected patients with low microfilaremia in Gabon, Central Africa, using the STARD-BLCM guidelines
Source: Parasit Vectors. 2024 Mar 15;17:138. doi: 10.1186/s13071-023-06089-1 (PMC10943916; doi:10.1186/s13071-023-06089-1)
Supplement: Supplementary file 2 — Additional file 2: Table S3. Criteria of a study conducted according to the Standards for the Reporting of Diagnostic Accuracy Studies that use Bayesian latent class models. [file 13071_2023_6089_MOESM2_ESM.docx]

**Additional file 2**

**Table S3:** Criteria of a study conducted according to Standards for the Reporting of Diagnostic accuracy studies that use Bayesian Latent Class Models

|  | **Section & Topic** | **No** | **Item** | **Reported on page #** |
| --- | --- | --- | --- | --- |
|  |  |  |  |  |
|  | **TITLE OR ABSTRACT** |  |  |  |
|  |  | **1** | Identification as a study of diagnostic accuracy, using at least one measure of accuracy (such as sensitivity, specificity, predictive values, or AUC) **and Bayesian latent class models** | 1, 3 |
|  | **ABSTRACT** |  |  |  |
|  |  | **2** | Structured summary of study design, methods, results, and conclusions  (for specific guidance, see STARD for Abstracts) | 3 |
|  | **INTRODUCTION** |  |  |  |
|  |  | **3** | Scientific and clinical background, including the intended use and clinical role of the **tests under evaluation** | 5 |
|  |  | **4** | Study objectives and hypotheses, **such as estimation of diagnostic accuracy of the tests for a defined purpose through BLCM** | 6 |
|  | **METHODS** |  |  |  |
|  | *Study design* | **5** | Whether data collection was planned before the **tests** were performed (prospective study) or after (retrospective study) | 6 |
|  | *Participants* | **6** | Eligibility criteria **and description of the source population** | 6 |
|  |  | **7** | On what basis potentially eligible participants were identified  (such as symptoms, results from previous tests, inclusion in registry) | 6 |
|  |  | **8** | Where and when potentially eligible participants were identified (setting, location and dates) | 6 |
|  |  | **9** | Whether participants formed a consecutive, random or convenience series | 6 |
|  | *Test methods* | **10** | **Description of the tests under evaluation**, in sufficient detail to allow replication, **and/or cite references** | 7, 8 |
|  |  | **11** | Rationale for choosing the **tests under evaluation in relation to their purpose** | 7 |
|  |  | **12** | Definition of and rationale for test positivity cut-offs or result categories of **the tests under evaluation**, distinguishing pre-specified from exploratory | 8 |
|  |  | **13** | Whether clinical information was available to the performers or readers of **the tests under evaluation** | 8 |
|  | *Analysis* | **14a** | **BLCM model** for estimating measures of diagnostic accuracy | 10 |
|  |  | **14b** | **Definition and rationale of prior information and sensitivity analysis** | 10 |
|  |  | **15** | How indeterminate results **of the tests under evaluation** were handled | N/A |
|  |  | **16** | How missing data **of the tests under evaluation** were handled | 9 |
|  |  | **17** | Any analyses of variability in diagnostic accuracy, distinguishing pre-specified from exploratory | 10 |
|  |  | **18** | Intended sample size and how it was determined | 9 |
|  | **RESULTS** |  |  |  |
|  | *Participants* | **19** | Flow of participants, using a diagram | Additional file 1: Fig S2 |
|  |  | **20** | Baseline demographic and clinical characteristics of participants | 11 |
|  |  | **21** | **Not applicable: the distribution of the targeted conditions is unknown, hence the use of BLCM** |  |
|  |  | **22** | Time interval and any clinical interventions between **the tests under evaluation** | 7, 8 |
|  | *Test results* | **23** | Cross tabulation of the **tests’ results (or for continuous tests results their distribution by infection stage)** | 27 |
|  |  | **24** | Estimates of diagnostic accuracy **under alternative prior specification** and their precision (such as 95% **credible/probability intervals**) | 12 |
|  |  | **25** | Any adverse events from performing **the tests under evaluation** | N/A |
|  | **DISCUSSION** |  |  |  |
|  |  | **26** | Study limitations, including sources of potential bias, statistical uncertainty, and generalisability | 15, 16 |
|  |  | **27** | Implications for practice, including the intended use and clinical role of **the tests under evaluation in relevant settings (clinical, research, surveillance etc.)** | 15, 16 |
|  | **OTHER INFORMATION** |  |  |  |
|  |  | **28** | Registration number and name of registry | N/A |
|  |  | **29** | Where the full study protocol can be accessed | N/A |
|  |  | **30** | Sources of funding and other support; role of funders | 18 |
|  |  |  |  |  |

STARD - BLCM

STARD-BLCM stands for “Standards for the Reporting of Diagnostic accuracy studies that use Bayesian Latent Class Models” and is a modification of the STARD statement (which was recently updated to STARD2015). STARD-BLCM aims to facilitate improved quality of reporting for diagnostic accuracy studies that use Bayesian latent class models in the absence of a reference standard. The proposed modifications are relevant to both Bayesian and frequentist estimation methods but the focus is on the former.

More information for STARD (STARD2015) can be found at: [http://www.equator-network.org/reporting-guidelines/stard](http://www.equator-network.org/reporting-guidelines/stard/)

More information for STARD-BLCM can be found at: [http://www.equator-network.org/reporting-guidelines/stard-blcm](http://www.equator-network.org/reporting-guidelines/stard-blcm/)
